# Acute pulmonary vein stenosis during radiofrequency catheter ablation for atrial fibrillation

**DOI:** 10.1002/joa3.12946

**Published:** 2023-10-21

**Authors:** Daisuke Yakabe, Masahiro Araki, Shujiro Inoue, Toshihiro Nakamura

**Affiliations:** ^1^ Department of Cardiovascular Medicine Clinical Research Institute, National Hospital Organization Kyushu Medical Center Fukuoka Japan

**Keywords:** atrial fibrillation, catheter ablation, complication, pulmonary vein stenosis

## Abstract

We encountered acute pulmonary vein (PV) stenosis during radiofrequency catheter ablation. PV stenosis was not apparent before redo ablation (A). However, acute PV stenosis was observed after repeat ablation, including carina ablation (B, C). Computed tomography performed 6 months post‐ablation revealed chronic PV stenosis (D).

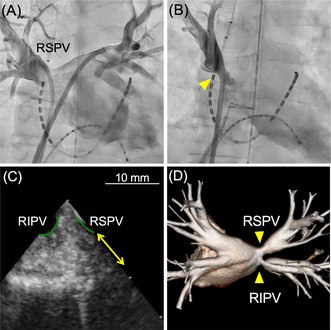

Pulmonary vein (PV) stenosis is a rare late‐onset complication after catheter ablation for atrial fibrillation (AF).^1^ Herein, we present a case of acute PV stenosis during ablation.

A male patient in his 60s and with no significant structural heart disease had undergone radiofrequency catheter ablation (PV isolation, including right PV carina ablation) for paroxysmal AF 18 months prior. However, he experienced AF recurrence after the index ablation, necessitating readmission for repeat ablation (second session). Computed tomography (CT) performed before the second session (Figure 1A) and left atrial angiography performed before ablation (Figure 1B, Video 1) showed mild right PV stenosis. Although we planned to perform repeat ablation for reconnection of the right superior PV, re‐isolation of the right superior PV was challenging even after multiple radiofrequency applications. A 50‐W power‐controlled mode was used, and the targeted ablation index was 450 with an irrigated ablation catheter (Thermocool Smarttouch SF D/D, Biosense Webster Inc., CA, USA). Considering the possibility of an epicardial connection, additional ablation of the PV carina was performed, which resulted in successful isolation (Figure 2). Subsequently, attempts to reinsert the decapolar ring catheter (20 mm Lasso catheter; Biosense Webster Inc.) into the right superior PV failed. Selective PV angiography revealed narrowing of the ostial right superior PV, which had not been observed before the ablation (Figure 3A, Video 2). Intracardiac echocardiography (Soundstar, Biosense Webster Inc.) revealed edematous walls of the right PVs and carina (Figure 3B,C). To evaluate whether this acute‐phase change was because of transient myocardial edema, CT was performed 6 months after ablation. The CT images demonstrated moderate stenosis in the right PVs (Figure 4A,B,C), with PV diameter reduction ratios of −71% and − 51% for the right superior and inferior PVs, respectively. The patient remained asymptomatic and was carefully followed‐up without invasive treatment.

**FIGURE 1:**
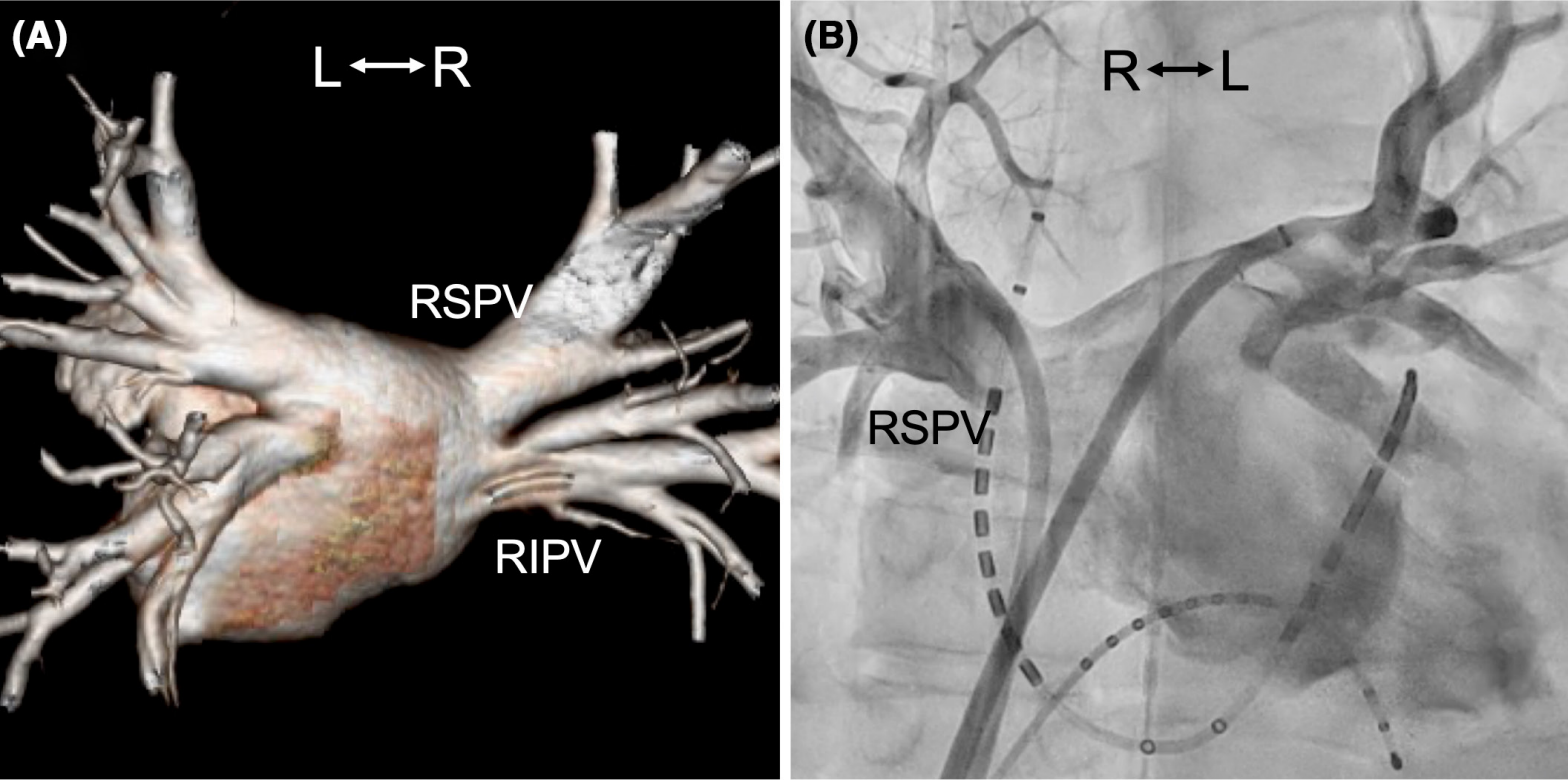
Computed tomography and left atrium angiography before ablation. The computed tomography image (A) and left atrium angiography (B) obtained before ablation demonstrated mild PV stenosis. (PV, pulmonary vein; RSPV, right superior PV; RIPV, right inferior PV).

**VIDEO 1:** To view this video in the full‐text HTML version of the article, please visit https://onlinelibrary.wiley.com/doi/10.1002/joa3.12946.

**FIGURE 2:**
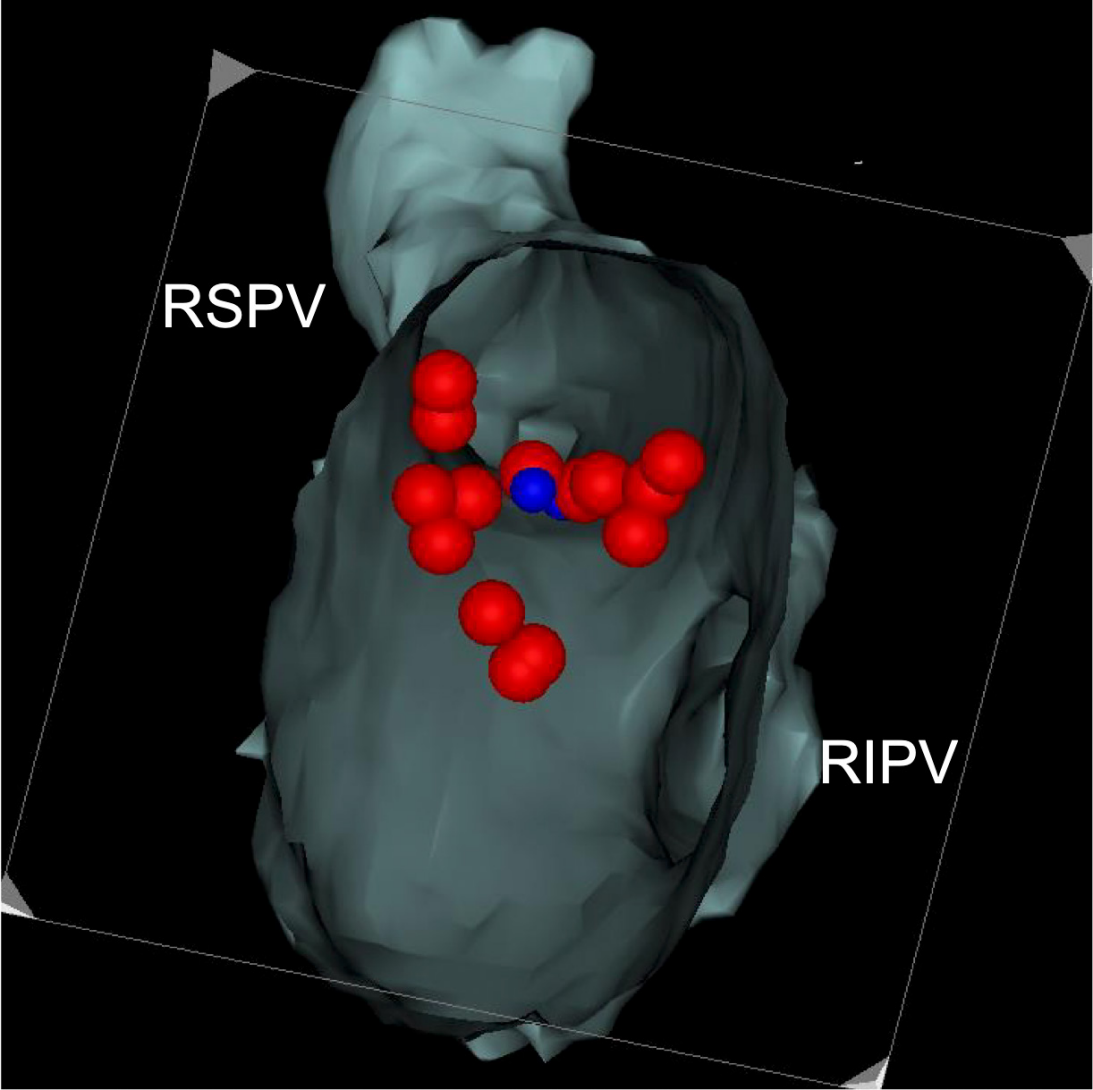
Successful ablation site of repeat PV isolation. Successful re‐isolation of RSPV was achieved while crossing the carina (blue point). Ablation power was controlled at 50 W, and targeted ablation index was 450. Total ablation time was 187 s (14 VisiTag points, represented in red when the targeted ablation index was reached).

**FIGURE 3:**
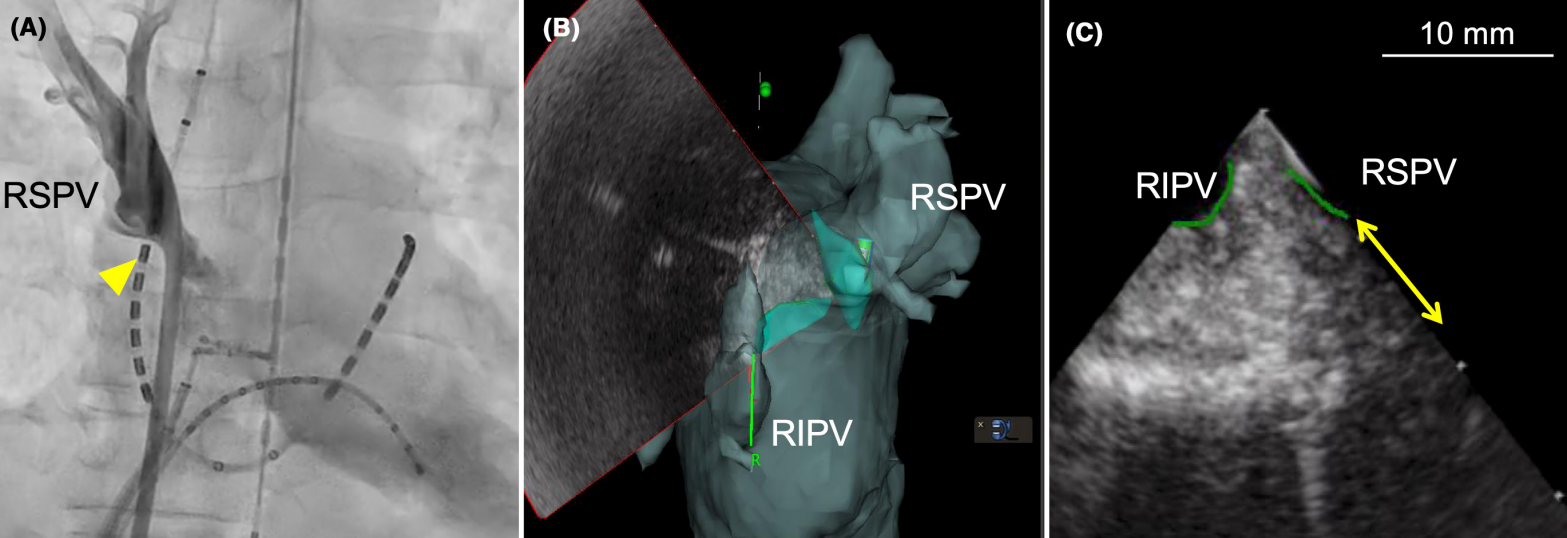
Acute PV stenosis after ablation. Selective PV angiography showed narrowing of RSPV. Intracardiac echocardiography demonstrated thickened and edematous wall of PVs.

**VIDEO 2:** To view this video in the full‐text HTML version of the article, please visit https://onlinelibrary.wiley.com/doi/10.1002/joa3.12946.

**FIGURE 4:**
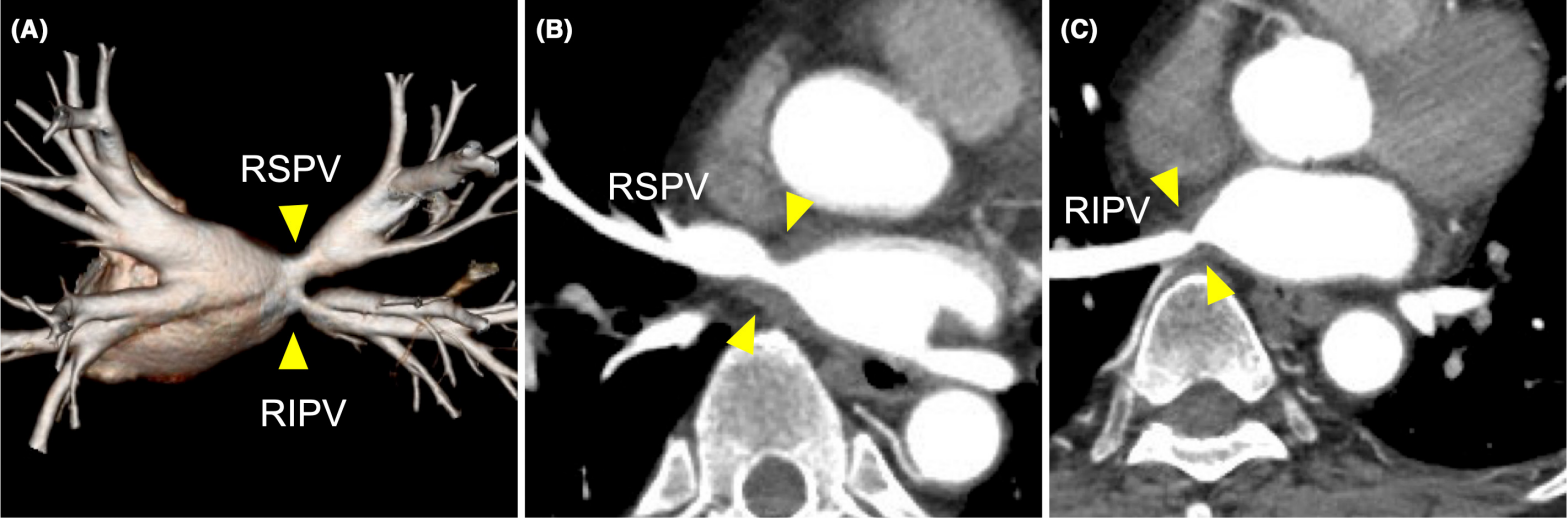
Computed tomography obtained 6 months after ablation. Chronic PV stenosis and thickened wall of ostial PVs were observed.

Acute PV narrowing or stenosis during ablation is rare. Typically, acute inflammatory responses and edematous changes in the PV wall after ablation tend to resolve spontaneously. According to a clinical study involving serial CT scans on the day after and 1 month after ablation, acute PV edema was frequently detected on the day after the procedure but disappeared after 1 month in all cases.^2^ However, in the present case, thickening of the PV wall and PV stenosis were observed even 6 months post‐ablation. The symptoms of PV stenosis generally become evident weeks to months following ablation and are often evaluated using CT at 3–6 months.^3^ Thus, we inferred that the acute‐phase edema had developed into chronic PV stenosis.

The optimal location of PV isolation line is a matter of concern. Recent literature has highlighted the presence of epicardial connections of PVs in certain cases, which has led to difficulties in successful PV isolation.^4^, ^5^ Ishikura et al. reported a low success rate of first‐pass isolation in cases with a broader isolation area encompassing an exit site in the left atrium of the epicardial connection, as identified by activation mapping during sinus rhythm.^4^ Although segmental ablation on the PV side facilitates complete PV isolation, it increases the risk of PV stenosis. Furthermore, the epicardial connection sites in the right PVs are frequently located in the carina, necessitating additional carina ablation.^5^ Although limited evidence indicates that adjunctive carina ablation poses a potential risk for chronic PV stenosis, we speculate that multiple radiofrequency applications, including carina ablation, may induce excessive inflammation and progress to fibrotic changes in the chronic phase. Further research is required to substantiate these hypotheses.

## CONFLICT OF INTEREST STATEMENT

The authors declare that they have no conflict of interest.

## ETHICS STATEMENT

The current study protocol was approved by the institutional review board of Kyushu Medical Center (23D059).

## PATIENT CONSENT STATEMENT

Written informed consent was obtained from the patient.

## Data Availability

All relevant datasets are within the manuscript.

## APPENDIX A

The videos of left atrial angiography before (Video 1) and after ablation (Video 2) are available.
